# Preconditioning with PDE1 Inhibitors and Moderate-Intensity Training Positively Affect Systemic Redox State of Rats

**DOI:** 10.1155/2020/6361703

**Published:** 2020-02-10

**Authors:** Jelena Ristic, Marko Folic, Katarina Radonjic, Milenko I. Rosic, Sergey Bolevich, Omarov Israpil Alisultanovich, Nevena Draginic, Marijana Andjic, Jovana Jeremic, Isidora Milosavljevic, Vladimir Zivkovic, Vladimir Jakovljevic

**Affiliations:** ^1^Representative Office Richter Gedeon Serbia, Belgrade, Serbia; ^2^University of Kragujevac, Faculty of Medical Sciences, Department of Pharmacy, Kragujevac, Serbia; ^3^Institute for Cardiovascular Diseases of Vojvodina, Clinic for Cardiovascular Surgery, Sremska Kamenica, Serbia; ^4^Faculty of Medicine, University of Novi Sad, Novi Sad, Serbia; ^5^1st Moscow State Medical University IM Sechenov, Department of Human Pathology, Moscow, Russia; ^6^Medical and Health Center of the Ministry of Foreign Affairs of Russia, Moscow, Russia; ^7^University of Kragujevac, Faculty of Medical Sciences, Department of Physiology, Kragujevac, Serbia

## Abstract

Taken into consideration that oxidative stress response after preconditioning with phosphodiesterase inhibitors (PDEIs) and moderate physical activity has still not been clarified, the aim of this study was to assess the effects of PDEIs alone or in combination with physical activity, on systemic redox status. The study was carried out on 96 male *Wistar albino* rats classified into two groups. The first group included animals exposed only to pharmacological preconditioning (PreC) maneuver (sedentary control (CTRL, 1 ml/day saline, *n* = 12), nicardipine (6 mg/kg/day of NIC, *n* = 12), vinpocetine (10 mg/kg/day of VIN, *n* = 12), and nimodipine (NIM 10 mg/kg/day of, *n* = 12). The second included animals exposed to preconditioning with moderate-intensity training (MIT) on treadmill for 8 weeks. After 5 weeks from the start of training, the animals were divided into four subgroups depending on the medication to be used for pharmacological PreC: moderate-intensity training (MIT+ 1 ml/day saline, *n* = 12), nicardipine (MIT+ 6 mg/kg/day of NIC, *n* = 12), vinpocetine (MIT+ 10 mg/kg/day of VIN, *n* = 12), and nimodipine (MIT+ 10 mg/kg/day of NIM, *n* = 12). After three weeks of pharmacological preconditioning, the animals were sacrificed. The following oxidative stress parameters were measured spectrophotometrically: nitrites (NO_2_^−^), superoxide anion radical (O_2_^−^), hydrogen peroxide (H_2_O_2_), index of lipid peroxidation (TBARS), superoxide dismutase (SOD), catalase (CAT), and reduced glutathione (GSH). Our results showed that PDE1 and MIT preconditioning decreased the release of prooxidants and improved the activity of antioxidant enzymes thus preventing systemic oxidative stress.

## 1. Introduction

Regular physical activity is considered to have various effects on different systems and organs as well as beneficial effects on lifestyle modifications. Therefore, it is seen as an indispensable element and a cornerstone in the nonpharmacological therapy of the cardiovascular, metabolic, and osteomuscular disorders [1]. Nowadays, scientists are engaged in finding the optimal intensity of physical activity in order to promote health and lifespan, improve quality of life, and decrease the incidence of lifestyle-related diseases [2, 3]. Moderate-intensity training (MIT) represents a training method involving longer-duration sessions of moderate-intensity exercise performed continuously without rest [4].

Based on epidemiological data, it has been observed that physical activity decreases the incidence of mortality caused by myocardial infarction; therefore, it is often studied as one of the nonpharmacological preconditioning (PreC) maneuvers [5, 6]. The mechanisms deemed to be responsible for the cardioprotective effects of physical activity have not yet been fully examined.

Besides nonpharmacological, various pharmacological PreC maneuvers have been extensively studied, but scientists have not yet been able to elucidate their complex cardioprotective effects [7, 8]. Controversial opinions and the literature date imply the role of various substances such as adenosine, norepinephrine, bradykinin, and free radicals and ATP-sensitive potassium channels in PreC. However, numerous investigations increasingly emphasize the role of calcium in both ischemia and PreC [9–11]. Ischemia has been repeatedly shown to reduce the available ATP, thereby inhibiting Na^+^-K^+^-ATP-ase thus resulting in calcium overload (*Ca*^2+^*overload*) [12]. Increased calcium production in the cytoplasm is significant in the myocardium injury as it enhances its binding to calmodulin kinase II and the formation of calcium/calmodulin (Са^2+^/СаМ) complex, activates nitric oxide synthase, which produces citrulline from L-arginine followed by nitrogen monoxide release [13].

One of the first research that gave the first insights in the association between oxidative status and training was implemented by Dillard and coworkers [14] which stimulated other researchers to investigate the role of reactive oxygen species (ROS) and other free radicals during physical activity. Under normal physiological conditions, prooxidants are continuously produced as a result of both, essential metabolic processes and environmental factors. However, previous data indicate that contrary to low physiological levels of prooxidants produced in the muscle which have an important role in normal contractility, excessive production of ROS leads to a contractile dysfunction followed by muscle weakness and exhaustion [15]. Preconditioning (hormesis) is a process by which small, nontoxic stresses are used to induce adaptive responses that protect biological systems against subsequently large and potentially lethal stresses of the same, similar, or different nature. The vast majority of observations revealed a hormetic dose response in the field of preconditioning/adaptive responses in animal studies. Furthermore, the elevated level of antioxidant system component reduced gluthathione (GSH), eliminates H_2_O_2_ leading to lower formation of ^·^OH and reduced polyunsaturated fatty acid peroxidation [16, 17].

A group of 11 enzyme (PDE1-11) families of metalophosphohydrolases and over 40 isoforms, called phosphodiesterases (PDEs) regulate the levels of cyclic guanosine monophosphate (cGMP) and cyclic adenosine monophosphate (cAMP), the second messengers in cell functions, by hydrolyzing these molecules. PDE inhibitors (PDEIs) have been attractive therapeutic targets due to their involvement in diverse medical conditions, e.g., cardiovascular, autoimmune diseases, dementia, etc. Furthermore, dihydropyridine derivatives have already been shown to have meaningly membrane biophysical interactions that lead to a potent lipid antioxidant effect independent of calcium channel modulation [18, 19].

Abovementioned facts suggest that the chronic use of Са^2+^/СаМ complex inhibitors (known as PDEIs) may be used as a pharmacological maneuver for PreC. Based on a previous study that was designed to examine the role of PDEIs in myocardial PreC, both alone and in combination with a nonpharmacological PreC maneuver. For nonpharmacological PreC, we chose physical activity according to the fact that trained rat hearts were less susceptible to calcium accumulation than the hearts of nontrained rats [20]. Since phosphodiesterase activity was noticed to be increased in monocytes and macrophages in response to proinflammatory stimuli such as histamine, lipopolysaccharide, and cytokines, the usage of PDE inhibitors might result in decreased production of ROS by macrophages and therefore alleviate oxidative stress [21].

Given the fact that oxidative stress response after preconditioning with PDEIs and moderate physical activity has not still been fully clarified, the aim of this study was to assess the effects of nicardipine, vinpocetine, and nimodipine alone or in combination with moderate physical activity, on systemic redox status in rats.

## 2. Material and Methods

### 2.1. Animals and Design of the Study

**Figure 1:**
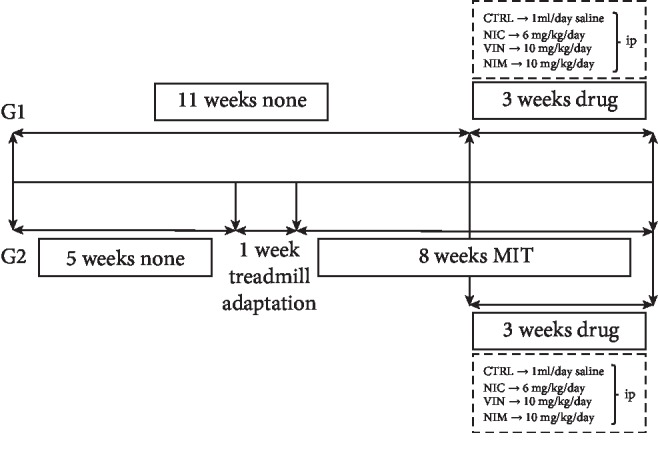
Design of the study and exercise protocol. G1—sedentary control group+drugs; G2—moderate-intensity training (MIT)+drugs. CTRL: control; NIC: nicardipine; VIN: vinpocetine; NIM: nimodipine.

According to the literature data and our previous investigations [4], we used ninety-six male *Wistar albino* rats that were kept on an artificial 12-h light-dark cycle (8 : 00 am–8 : 00 pm) at room temperature (22 ± 2°C). Water and food were available *ad libitum*. The animals were housed in their respective groups in a collective cage and received water and standard laboratory chow. The animals were divided in two groups (Figure 1).

The first group (11 weeks old at the beginning of experiments, *n* = 48), body weight: 270 ± 50 g, included animals exposed only to pharmacological preconditioning maneuver (i.p. injection of a suitable phosphodiesterase 1 inhibitor for 3 weeks). Depending on the pharmacological agent used, it was divided into four subgroups: sedentary control (CTRL, 1 ml/day saline, *n* = 12), nicardipine (6 mg/kg/day of NIC, *n* = 12), vinpocetine (10 mg/kg/day of VIN, *n* = 12), nimodipine (NIM 10 mg/kg/day of, *n* = 12).

The second group (6 weeks old at the beginning of experiments, *n* = 48), body weight: 270 ± 50 g, included animals exposed to PreC with physical activity for 8 weeks. After five weeks from the start of physical activity, the animals were divided into four subgroups depending on the medication to be used for pharmacological PreC: moderate-intensity training (MIT+ 1 ml/day saline, *n* = 12), nicardipine (MIT+6 mg/kg/day of NIC, *n* = 12), vinpocetine (MIT+10 mg/kg/day of VIN, n = 12), nimodipine (MIT+10 mg/kg/day of NIM, *n* = 12). After three weeks of pharmacological preconditioning, the animals were sacrificed.

PDE 1 inhibitor drugs were dissolved in dilute dimethyl sulfoxide (DMSO) solution (DMSO: saline (10 : 90)) [22]. The same amount of DMSO will be applied in the control groups.

### 2.2. Compliance with Ethical Standards

This research was carried out in the Laboratory for Cardiovascular Physiology of the Faculty of Medical Sciences, University of Kragujevac, Serbia. The study protocol was approved by the Ethical Committee for the welfare of experimental animals of the Faculty of Medical Sciences, University of Kragujevac, Serbia. All experiments were performed according to EU Directive for welfare of laboratory animals (86/609/EEC) and principles of Good Laboratory Practice.

### 2.3. Exercise Protocol

Exercise protocol was performed by Treadmill for rats (ELUNIT Medical Equipment), customized for anatomical and physiological characteristics of small experimental animals (power supply 220 V, 50 Hz, number of trails for running: 4; speed control 2–50 m/min with a resolution of 0.1 m/min, bars which deliver mild electric shock of 0–0.5 mA with a 1–2 s interpulse interval), connected with Treadmill software to monitor speed continuously. Mild electric shock was activated when rats stop running but without causing stress to animals [23].

In our study, moderate-intensity training (MIT) was performed (Figure 1 and Table 1). The rats on exercise MIT protocol ran on treadmill for 8 weeks-5 days, with 1 week of adaptation period before (8 m/s speed for 30 min/day), and with gradual increase in speed during weeks, from 10 m/min in the second week to 15 m/min in the eighth week with 3 min rest/100 m and 5 min warmup at 8 m/min, prior to each training session [4].

### 2.4. Blood Sample Collection

After 48 h of rest following the final training, the rats were fasted for 24 h and sacrificed by decapitation after short-term narcosis induced by intraperitoneal application of ketamine (10 mg/kg) and xylazine (5 mg/kg) and premedication with heparin as an anticoagulant whereby blood samples were collected. A blood sample were collected into ice-cold EDTA capillary system tubes by the nick procedure, and approximately 500 *μ*l of blood plasma sample obtained after centrifugation and was stored at −80°C until use for biochemical analysis.

### 2.5. Evaluation of Systemic Redox State

In the moment of sacrificing animals, blood samples were collected from the jugular vein in order to estimate following prooxidants in serum: the levels of nitrites (NO_2_^−^), superoxide anion radical (O_2_^−^), hydrogen peroxide (H_2_O_2_), index of lipid peroxidation (thiobarbituric acid reactive substances, TBARS), and parameters of antioxidative defense system in erythrocyte samples: superoxide dismutase (SOD), catalase (CAT), and level of reduced glutathione (GSH).

### 2.6. Markers of Oxidative Stress

Nitric oxide decomposes rapidly to form stable metabolite nitrite/nitrate products. Nitrite level (NO_2_^−^) was measured spectrophotometrically at a wavelength of 543 nm and used as an index of nitric oxide (NO) production using the Griess's reagent as previously described by Green and coworkers [24]. Measurement of hydrogen peroxide (H_2_O_2_) is based on oxidation of phenol red by hydrogen peroxide, in a reaction catalyzed by horseradish peroxidase (HRPO) at 610 nm, as previously described by Pick and Keisari [25]. Index of lipid peroxidation (thiobarbituric acid reactive substances (TBARS)) was estimated by measuring of TBARS using 1% TBA (thiobarbituric acid) in 0.05 NaOH incubated with the plasma as previously described [26]. The concentration of superoxide anion radical (O_2_^−^) was measured by NBT (nitro bluetetrazolium) reaction in TRIS buffer with plasma sample at 530 nm as previously described by Auclair and Voisin [27]. All analysis was determined using the spectrophotometrical method (UV-1800 UV–Vis Spectrophotometer by Shimadzu Scientific Instruments Inc.).

### 2.7. Antioxidative Enzymes

For determination of antioxidant parameters, isolated erytrocytes were prepared according to McCord and Fridovich [28]. SOD activity was determined by the epinephrine method described by Misra and Fridovich. A 100 *μ*l lysate and 1 ml carbonate buffer were mixed, and then 100 *μ*l of epinephrine was added. Detection was performed at 470 nm [29]. CAT activity was determined according to Beutler. Lysates were diluted with distilled water (1 : 7 *v*/*v*) and treated with chloroform-ethanol (0.6 : 1 *v*/*v*) to remove hemoglobin and then, 50 *μ*l CAT buffer, 100 *μ*l sample, and 1 ml 10 mM H_2_O_2_ were added to the samples. Detection was performed at 360 nm [30]. The level of reduced glutathione (GSH) was determined based on GSH oxidation with 5.5-dithio-bis-6.2-nitrobenzoic acid, as previously described by Beutler. Measuring was performed at 420 nm [31].

### 2.8. Statistical Analysis

IBM SPSS Statistics 20.0 Desktop for Windows was used for statistical analysis. Distribution of data was checked by Shapiro–Wilk test. Where distribution between groups was normal, statistical comparisons were performed using the one-way analysis of variance (ANOVA) tests with a Tukey's post hoc test for multiple comparisons. Kruskal–Wallis was used for comparison between groups where the distribution of data was different than normal. Values of *p* < 0.05 were considered to be statistically significant.

## 3. Results

### 3.1. Hydrogen Peroxide Determination (H_2_O_2_) and Superoxide Determination (O_2_^−^)

**Figure 2:**
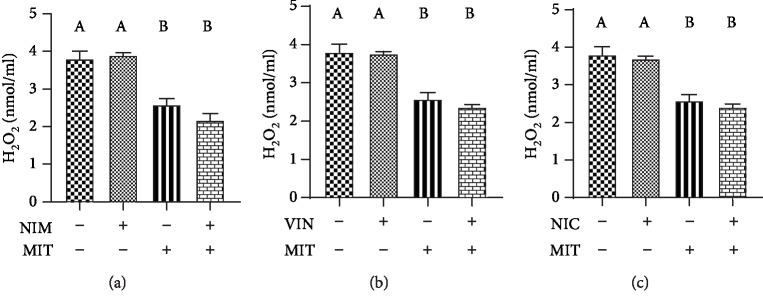
Effects of exercise and PDE-1 inhibitors on the level of hydrogen peroxide determination (H_2_O_2_). (a) nimodipine (NIM). (b) vinpocetine (VIN). (c) nicardipine (NIC). MIT: moderate-intensity training. The same letter means that there is no significant difference; not sharing the same letter means that there is significant difference. Data are means ± SD (*n* = 12 per group).

**Figure 3:**
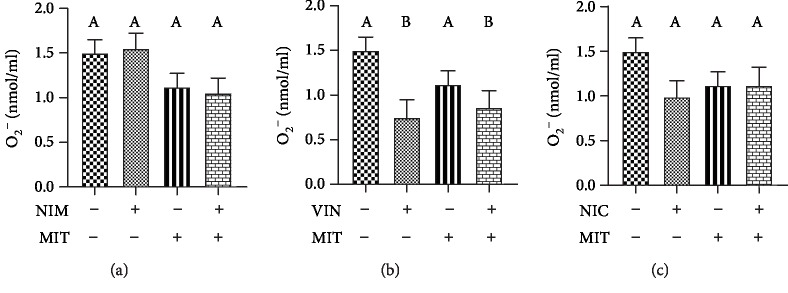
Effects of exercise and PDE-1 inhibitors on the level of superoxide anion radical (O_2_^−^). (a) nimodipine (NIM). (b) vinpocetine (VIN). (c) nicardipine (NIC). MIT: moderate-intensity training. The same letter means that there is no significant difference; not sharing the same letter means that there is significant difference. Data are means ± SD (*n* = 12 per group).

Level of H_2_O_2_ was significantly lower in the training group than in the control group. Training as well as combination of training and drug (NIM +MIT group, VIN+MIT group, NIC+MIT group) had significantly diminishing effect on level of H_2_O_2_ relative to all examined drugs (nimodipine, vinpocetine, and nicardipine) as well as to the control group (Figure 2). The O_2_^−^ level significantly decreased after vinpocetine application compared to the control group. Additionally MIT+VIN group reduced level of O_2_^−^ relative to the control group (Figure 3).

### 3.2. Nitrite Determination (NO_2_^−^)

**Figure 4:**
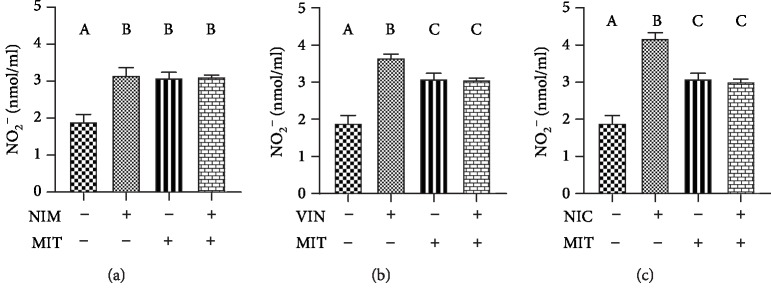
Effects of exercise and PDE-1 inhibitors on the level of nitrite (NO_2_^−^). (a) nimodipine (NIM). (b) vinpocetine (VIN). (c) nicardipine (NIC). MIT: moderate-intensity training. The same letter means that there is no significant difference; not sharing the same letter means that there is significant difference. Data are means ± SD (*n* = 12 per group).

Level of NO_2_^−^ was significantly increased after training, application of drugs (nimodopine, vinpocetine, and nicardipine) as well as after combination of drug and training (NIM+MIT group, VIN+MIT group, NIC+MIT group) compared to the control group. The training group had significantly lower level of NO_2_^−^ relative to the group receiving vinpocetine or nicardipine. In the VIN+MIT group and NIC+MIT group, we observed significantly decreased level of nitrite compared to VIN and NIC group, respectively (Figure 4).

### 3.3. Index of Lipid Peroxidation Measured as TBARS

**Figure 5:**
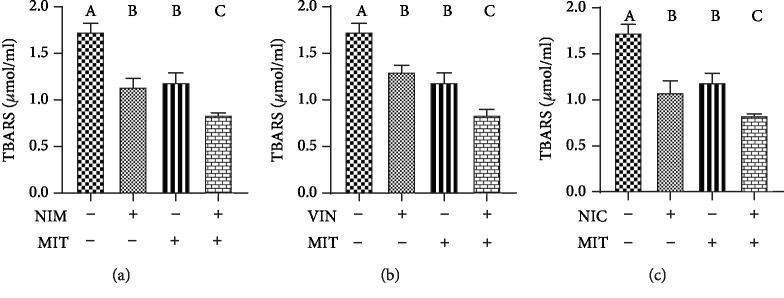
Effects of exercise and PDE-1 inhibitors on the level of TBARS. (a) nimodipine (NIM). (b) vinpocetine (VIN). (c) nicardipine (NIC). MIT: moderate-intensity training (MIT). The same letter means that there is no significant difference; not sharing the same letter means that there is significant difference. Data are means ± SD (*n* = 12 per group).

The TBARS concentration was significantly decreased after training compared to the control group. Vinpocetine, nimodipine, and nicardipine significantly reduced TBARS level relative to the control group. Combination of drug and training (NIM+MIT group, VIN+MIT group, NIC+MIT group) lead to significant decrease of TBARS concentration in comparison to the control as well as the training group. Furthermore, values of TBARS concentration were significantly higher in the vinpocetine, nimodipine, and nicardipine groups relative to VIN+MIT, NIM+MIT, and NIC+MIT groups, respectively (Figure 5).

### 3.4. Catalase (CAT), Superoxide Dismutase (SOD), and Reduced Glutathione (GSH)

**Figure 6:**
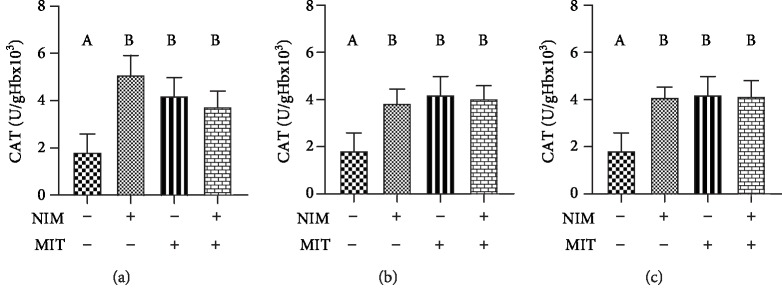
Effects of exercise and PDE-1 inhibitors on catalase (CAT) activity. (a) nimodipine (NIM). (b) vinpocetine (VIN). (c) nicardipine (NIC). MIT: moderate-intensity training. The same letter means that there is no significant difference; not sharing the same letter means that there is significant difference. Data are means ± SD (*n* = 12 per group).

**Figure 7:**
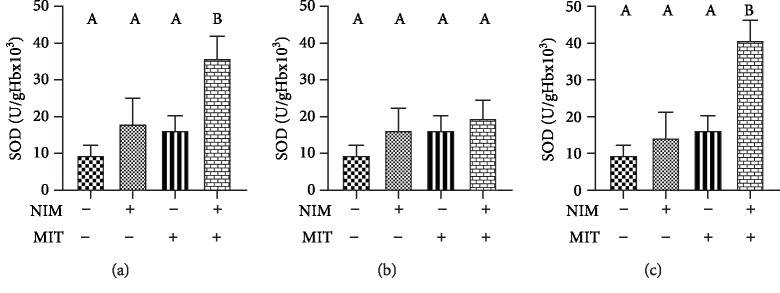
Effects of exercise and PDE-1 inhibitors on superoxide dismutase (SOD) activity. (a) nimodipine (NIM). (b) vinpocetine (VIN). (c) nicardipine (NIC). MIT: moderate-intensity training. The same letter means that there is no significant difference; not sharing the same letter means that there is significant difference. Data are means ± SD (*n* = 12 per group).

**Figure 8:**
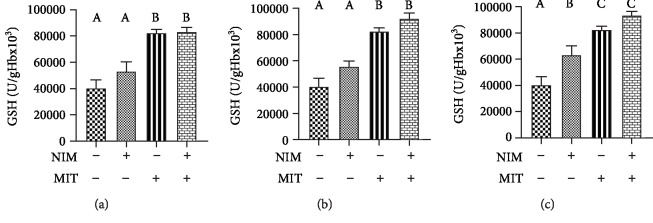
Effects of exercise and PDE-1 inhibitors on the level of reduced glutathione (GSH). (a) nimodipine (NIM). (b) vinpocetine (VIN). (c) nicardipine (NIC). MIT: moderate-intensity training (MIT). The same letter means that there is no significant difference; not sharing the same letter means that there is significant difference. Data are means ± SD (*n* = 12 per group).

Activity of enzyme CAT significantly increased after training protocol relative to the control group. All examined PDE-1 inhibitors showed increased activity of CAT compared to the control group. Combination of exercise and examined drugs (nimodipine, vinpocetine, and nicardipine) induced higher CAT activity relative to the control group (Figure 6). Combination of training and PDEIs (nimodipine and nicardipine) led to increased activity of SOD enzyme (Figure 7). The level of reduced glutathione was increased after exercise and nicardipine application relative to the control group. Training as well as all PDE1 inhibitors (nimodipine, vinpocetine, and nicardipine) in combination with training significantly increased level of GSH compared with PDE1 inhibitors applied alone (Figure 8).

## 4. Discussion

In order to achieve cardioprotection, a possible approach might lie in the prevention of excessive accumulation of intracellular calcium. Regarding the protective effect of physical activity on the heart, we aimed to estimate the potential benefits of MIT combined with nimodipine, vinpocetine, and nicardipine on systemic oxidative stress. We have chosen nimodipine, vinpocetine, and nicardipine as calcium channel modulators and PDE1 inhibitors with an antioxidant property [32].

Results of our study clearly indicate that nimodipine, nicardipine, and training alone and their combination did not affect plasma level of superoxide anion radical of examined rats. On contrary, our previous investigation established that during both MIT and HIT protocols the level of O_2_^−^ significantly increased relative to the control normotensive group of rats [4]. The reason for different levels of O_2_^−^ after training might be the duration of exercise protocols. It seems that eight weeks of moderate running training do not promote oxidative damage or act protectively within the heart. Opposite to our data, earlier investigation conducted on human and rabbit neutrophils indicate that dihydropyridines, especially nicardipine inhibit superoxide anion radicals by blockage of cytosolic Ca^2+^ mobilization and protein kinase C activation [33]. On the other hand, level of O_2_^−^ decreased after vinpocetine alone or its combination with training. These results are consistent with an earlier study which showed that vinpocetine in a dose of 30 mg/kg reduced superoxide anion which increased after carrageenan administration [34]. In the same study, it had been shown that neutrophils are cells that are an important source of superoxide anion. Vinpocetine reduced neutrophil recruitment thus reduces the level of the superoxide anion and has an antioxidant effect [34].

With regard to concentration of H_2_O_2_, our results revealed decreased level of that prooxidant after training but not after all applied PDE-1 inhibitors (nimodipine, vinpocetine, and nicardipine). Combination of exercise and all PDE-1 inhibitors induced reduction in H_2_O_2_ which we can attribute to exercise since calcium blockers did not affect H_2_O_2_ alone. Potential explanation for diminished plasma concentration of H_2_O_2_ during exercise may be due to enhanced CAT activity which catalyzed reaction of H_2_O_2_ degradation to oxygen and water. Furthermore, the elevated level of antioxidant system component reduced glutathione (GSH), eliminated H_2_O_2_ leading to lower formation of ^·^OH and reduced polyunsaturated fatty acid peroxidation [35].

It is already established that treadmill exercise involves increased the gene expression of eNOS and enhanced production of NO [36]. Furthermore, research in a field of calcium blockers showed that these drugs are responsible for significant level of nitrite monoxide in the endothelium [37, 38]. Bearing in mind that half-life of NO is very short, our study focused on active metabolites of NO such as nitrites. In our investigation, we observed that level of nitrites after treadmill exercise was increased. Interestingly, nicardipine and its combination with training also lead to significantly high level of NO_2_^−^. In addition, these results are in keeping with the findings of several investigators. Single application of nicardipine to rabbits led to increased production of nitrites [39]. Moreover, nicardipine prevented enhanced NO degradation without affecting superoxide dismutase and catalase activities [40]. Dual mechanism of nicardipine action as calcium channel and PDE inhibitor may describe higher production of NO. PDE1 inhibition leads to hypotension by high amount of NO due to the reaction of hydrolyzation of cAMP and cGMP [41].

Since cytosolic variation of calcium level may cause lipid peroxidation, many investigations have been performed in terms of prevention of endothelial cell damage. Measuring index of lipid peroxidation, we revealed that all PDE-1 inhibitors (nimodipine, vinpocetine, and nicardipine) as well as training decreased TBARS level. Furthermore, application of both maneuvers led to a reduction of TBARS. Our results are in accordance with previous investigation. TBARS levels measured as MDA showed significant decrease after pretreatment with nimodipine and vinpocetine or combination of these drugs compared to the group with myocardium infarction, indicating the antioxidative role of these drugs [32]. Also, vinpocetine improved oxidative stress in gentamicin-induced acute kidney injury through the reduction of MDA serum level [42]. The efficacy of vinpocetine, in inhibiting lipid peroxidation, correlates well with their protective actions against glutamate-induced cytotoxicity [43]. It is believed that nicardipine can prevent the formation of atherosclerotic plaques which is accompanied by reduction of lipid peroxidation [44]. In the model of experimental subarachnoid hemorrhage rats, nicardipine reduced index of lipid peroxidation measured as TBARS; however, it did not affect MDA production [45]. Nevertheless, MDA and NADPH oxidase levels were decreased by nicardipine in hypertensive rats reflecting antioxidant properties of nicardipine [46]. However, even the determination of TBARS is most frequently used, and is a convenient and simple method, there is a drawback with specificity and variability of data. Furthermore, we should bear in mind that MDA (measured through the spectrophotometric TBARS assay) is a measure of oxidative damage on lipids, not of “oxidative stress” *per se* [47, 48]. On the other hand, 15-F2t-Isoprostane represents a specific, quantitative parameter of oxidative stress *in vivo* which may be the issue of our future research [49].

Observing the antioxidative enzymes, PDE1 inhibitors showed positive effect on this system. We reported increased level of CAT after training, all PDE-1 inhibitors and combination of training and examined drugs. In measuring activity of SOD, high values were only noticed after combination of training and PDE-1 inhibitors (nimodipine and nicardipine). Additionally, the level of GSH showed significant increase after exercise and both exercise and PDE-1 inhibitors. Nicardipine alone compared to the control group point out a significant increase in the concentration of this parameter. Our data corroborate other reports which showed that treatment with vinpocetine and nimodipine alone or in combination increased the activities of GSH, CAT, and SOD in isoproterenol-induced myocardial infarction in rats. Thus, this antioxidant effect accounts for the cardioprotective property of vinpocetine and nimodipine [32]. Interestingly, in the case of vinpocetine, with the higher dose of vinpocetine, the increment in GSH was less apparent in the hippocampus and striatum. An intriguing explanation for these observations is that at its high concentration, vinpocetine exhibit prooxidant properties and increase free radical production or act as a free radical [50]. As mentioned above, GSH is responsible for lowering the H_2_O_2_ level. Earlier research estimated that calcium channel blockers such as nicardipine showed antioxidant properties by prevention of GSH loss [33]. On the other hand, a scientist observed that the elevated level of GSH in spontaneously hypertensive rats, caused by enhanced activity of xanthine oxidase, can be normalized by application of nicardipine twenty years ago [51]. One of the possible explanation for the antioxidant activity of all three PDE-1 inhibitors may be high lipophilicity and the chemical structure of those drugs. Actually, they belong to group of dihydropyridine derivatives involved in the electron transfer process and biophysical interactions causing antioxidant properties apart from the effect on calcium channel [52].

## 5. Conclusion

In summary, our results pointed out that moderate-intensity physical training of sufficient duration alone or in combination with PDE-1 inhibitors leads to the beneficial adaptations, manifested as decreased oxidative stress and improved antioxidant defense system.

In addition, these findings can lead to both, developing of physical and pharmacological strategies to prevent oxidative stress and in the design of therapeutic strategies for treating relevant, oxidative stress-induced disorders in humans. Further investigations should be conducted to clarify the exact mechanism of the PDE1 inhibitor and physical activity protective effects.

## Figures and Tables

**Table 1:** Moderate-intensity training protocol.

Weeks/days^∗^/^∗∗^	Monday	Tuesday	Wednesday	Thursday	Friday
Adaptation	8 m/min for 30 min				
1	10 m/min for 1 h	10 m/min for 1 h	10 m/min for 1 h	10 m/min for 1 h	10 m/min for 1 h
2	10 m/min for 1 h	10 m/min for 1 h	10 m/min for 1 h	10 m/min for 1 h	10 m/min for 1 h
3	11 m/min for 1 h	11 m/min for 1 h	11 m/min for 1 h	11 m/min for 1 h	11 m/min for 1 h
4	12 m/min for 1 h	12 m/min for 1 h	12 m/min for 1 h	12 m/min for 1 h	12 m/min for 1 h
5	12 m/min for 1 h	12 m/min for 1 h	12 m/min for 1 h	12 m/min for 1 h	12 m/min for 1 h
6	13 m/min for 1 h	13 m/min for 1 h	13 m/min for 1 h	13 m/min for 1 h	13 m/min for 1 h
7	14 m/min for 1 h	14 m/min for 1 h	14 m/min for 1 h	14 m/min for 1 h	14 m/min for 1 h
8	15 m/min for 1 h	15 m/min for 1 h	15 m/min for 1 h	15 m/min for 1 h	15 m/min for 1 h

^∗^3 min rest/100 m. ^∗∗^5 min warmup at 8 m/min prior to each training session.

## Data Availability

The data used to support the findings of this study are available from the corresponding author upon request.
